# Loss of Infectivity of HIV-1 Particles Produced by Mobile Lymphocytes

**DOI:** 10.1371/journal.pone.0109601

**Published:** 2014-10-08

**Authors:** Maxime Chazal, Patrycja Nzounza, Claudine Pique, Bertha Cecilia Ramirez

**Affiliations:** 1 INSERM, U1016, Institut Cochin, Paris, France; 2 CNRS, UMR8104, Paris, France; 3 Université Paris Descartes, Sorbonne Paris Cité, Paris, France; Institut National de la Santé et de la Recherche Médicale, France

## Abstract

HIV-1 spreads by cell-free particles and through direct cell contacts. To discriminate between these two modes of dissemination, an assay in which the cells are cultured under shaking conditions impairing cell-to-cell transmission has been described. We addressed the impact of shaking on HIV-1 particle infectivity. Kinetics of HIV-1 infection in static or shaking conditions confirmed that HIV-1 replication is reduced in mobile lymphocyte T cells. Strikingly, the infectivity of viruses produced by mobile lymphocytes was dramatically reduced. In parallel, the amount of envelope protein present on these particles showed a continuous decrease over time. We conclude that inefficient HIV-1 replication in mobile lymphocytes in this experimental system is not only due to avoidance of viral cell-to-cell transfer but also to the loss of infectivity of the viral particles due to the alteration of the composition and functionality of the particles produced by these lymphocytes. It is important to take these observations into account when studying viral transmission under shaking conditions.

## Introduction

HIV-1 replication and transmission is commonly assessed *in vitro* in static cell cultures [1], [2], [3]. Sourisseau *et al.* proposed that this assay did not represent the situation encountered by lymphocytes in fluids and established an experimental system of continuously shaking cultures to mimic the infection of mobile lymphocytes [4]. The authors compare HIV-1 replication kinetics in static and continuously shaking lymphocyte cultures, they conclude that shaking culture conditions prevent cell contacts, thus avoiding virus transfer through direct cell contacts. This system of shaking culture is widely used to study differences between cell-free and cell-to-cell HIV-1 transmission [5], [6], [7]. Here we show that shaking culture of HIV-1-infected T cells not only avoids cell contacts preventing the transfer of virus from cell to cell but, after 24 hours, it also affects cell-free virus transmission by inducing loss of HIV-1 infectivity and reduction of envelope proteins from the surface of the viral particles.

## Materials and Methods

### Cells and cell culture

CD4+/CXCR4+ Jurkat T cells (clone 20; a kind gift of Dr. Olivier Schwartz, Institut Pasteur, Paris, France) were maintained in complete RPMI medium: RPMI 1640 (Gibco) supplemented with 10% FCS, streptomycin (100 mg/mL; Gibco), penicillin (100 U/mL; Gibco), glucose (0.43%, Gibco) and glutamine (2 mM; Gibco). CD4+/CXCR4+ Jurkat T cells were cultured at 37°C under static or gentle shaking conditions as described previously (SpeciMix; Bioblock Scientific, 40 movements/min) [4].

293 T and HeLa P4.2 reporter cells (Hela-CD4-HIV-LTR-lacZ cells) were maintained in DMEM medium (Gibco) supplemented with 10% FCS, streptomycin (100 mg/mL), penicillin (100 U/mL) and glutamine (2 mM).

### HIV-1 infection

The X4 NL4.3 strain of HIV-1 was produced in 293 T cells (1.5×10^6^) transfected with 5 µg of pNL4.3 proviral plasmid (obtained from the NIH AIDS Research and Reference Reagent Program) by the calcium phosphate technique and supernatants of cultured cells were collected 48 and 72 h post-transfection. A minimum of ten million of Jurkat T cells were infected with HIV-1 NL4.3 at a multiplicity of infection (MOI) of 0.001 in complete RPMI medium during two hours at 37°C, the viral inoculum was then washed off with RPMI and cells were cultured at 37°C under static or gentle shaking conditions.

Kinetics of infection were followed by determining the fraction of HIV-1-infected cells in the T cell cultures by measuring the percentage of Gag p24+ cells by flow cytometry after Gag labeling with the anti-HIV-p24 KC57-PE monoclonal antibody (1/500; Coulter Beckman; mAb) followed by cytometry analysis (Canto 2 cytometer or FC-500 Cytomics) as reported previously [8]. The cells were fixed with 4% paraformaldehyde, washed with PBS buffer containing 2% BSA and 0.1% Tween 20 and stained with the KC57-PE mAb (Coulter Beckman) which recognizes the 55, 39, 33 and 24 kDa proteins of the core of HIV-1.

The cell-surface level of the HIV-1 envelope was measured by flow cytometry using the anti-Env 5F7 mAb (AIDS Research and Reference Reagent Program) and the PE-conjugated secondary Ab (Dako). Tubulin levels were measured by using the anti-tubulin mAb (Sigma-Aldrich).

### Infectivity test of HIV-1 particles

HIV-1 p24 content was determined using the ELISA INNOTEST HIV (INGEN). Equal amounts of virus (from 1 to 5 ng of HIV-1 p24) were used to infect HeLa P4.2 reporter cells. After 36 h of incubation, the cells were lysed and β-galactosidase production was assessed by a colorimetric assay [8] based on cleavage of chlorophenol red-β-D-galactopyranoside (CPRG).

### Analysis of HIV-1 particles

Particles were collected from supernatants of infected cultures, filtered (0.45 µm) and ultracentrifuged through a 25% sucrose cushion in TNE buffer (10 mM Tris-HCl pH 7.4, 100 mM NaCl, and 1 mM EDTA). Ultracentrifugation was performed at 150 000×g for 1 h at 4°C in a Beckman SW41 Ti rotor and viral pellets were resuspended in 30 µL lysis buffer (20 mM Tris-HCl pH 8, 0.2 mM EGTA, 120 mM NaCl, 0.2 mM NaF, 0.2% sodium deoxycholate, 0.5% NP40, and complete protease inhibitors; Roche Applied Science) before polyacrylamide gel separation and immunoblotting.

The levels of the HIV-1 proteins gp120, gp41, Gag Pr55 and Gag p24 were estimated using respectively the following primary anti-gp120 goat polyclonal Ab (Abcam), HIV-1 gp41 mAb (2F5; AIDS Research and Reference Reagent Program) and anti-CA-p24 #24-2 mAb (AIDS Research and Reference Reagent Program).

### Statistical analysis

Statistical analyses were performed using the Mann Whitney test. Statistical significance was defined for P values<0.05.

## Results

### Infectivity of HIV-1 cell free particles is reduced in mobile lymphocyte T cells

**Figure 1:**
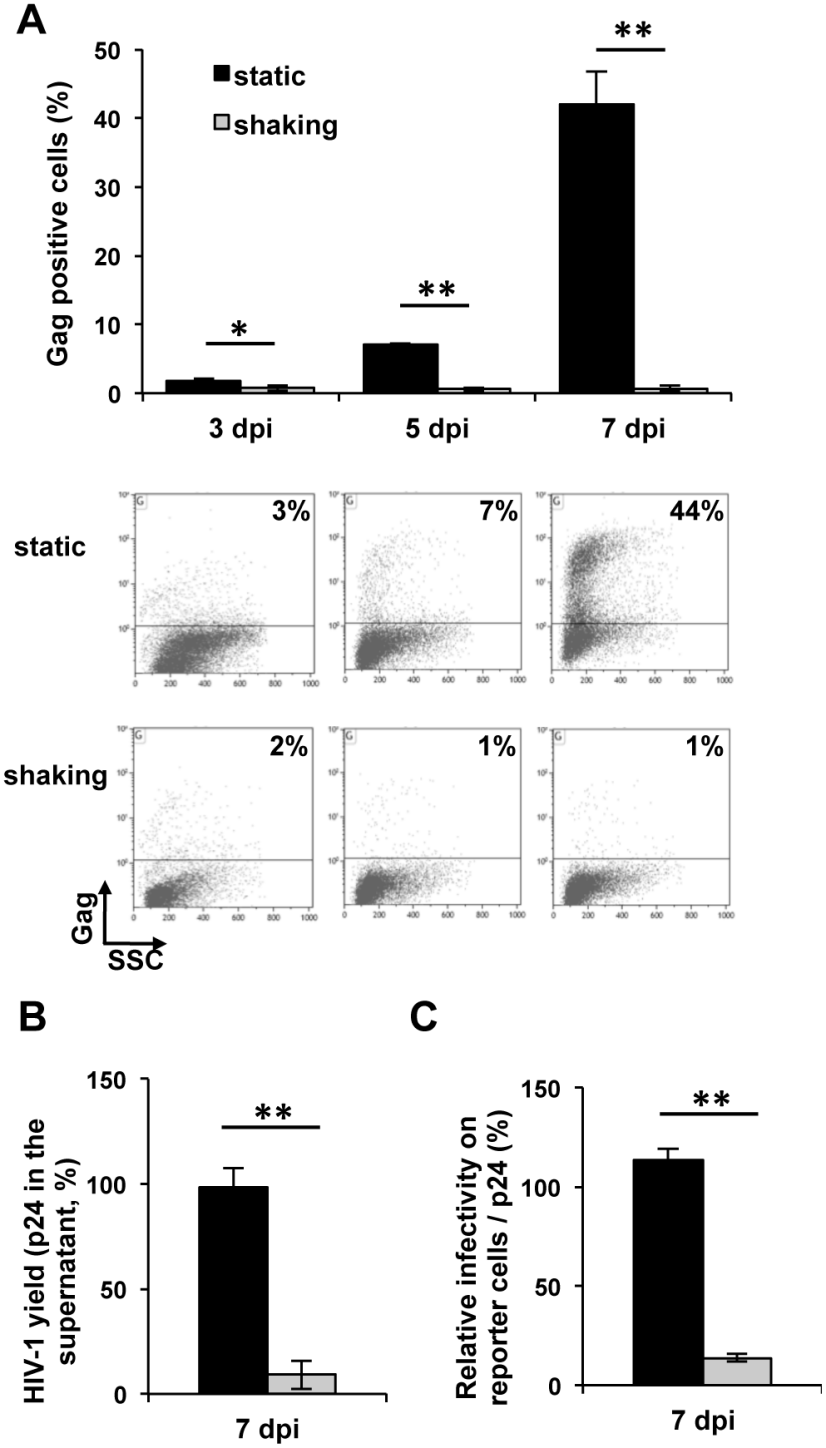
HIV-1 replication and particle infectivity are drastically reduced in mobile lymphocyte T cells. **A.** HIV-1 replication in Jurkat T cells cultured under static or shaking conditions. Jurkat T cells infected with the NL4.3 HIV strain were cultured under static or shaking conditions. Viral replication was measured by determining the fraction of HIV-1-infected cells in the two cultures by intracellular Gag labeling and flow cytometry. Cells were labeled with the anti-HIV-p24 mAb KC57-PE, 3, 5 and 7 days post infection (dpi). Data in top pannel are the means of three independent experiments performed in duplicate. FACS profiles in bottom pannel are of one representative experiment. P = 0.01 for 3 dpi. P = 0.006 for 5 dpi. P = 0.005 for 7 dpi. Error bars represent standard error of the mean (SEM). *, P<0.05. **, P<0.01. **B. HIV-1 yield of Jurkat T cells cultured under static or shaking conditions.** Supernatants were collected at 7 dpi, filtered and the p24 content was measured by ELISA. The values were normalized for protein content of extracts of cultured cells. The data are means of three independent experiments each carried out in duplicate. For each experiment the values were normalized taking as 100% the value obtained for one of the duplicates of cells under static culture at 7 dpi. P = 0.003 at 7 dpi. **C. Infectivity of HIV-1 particles produced by Jurkat T cells cultured under static or shaking conditions.** Viral supernatants of Jurkat T cells infected with HIV-1 NL4.3 were collected at 7 dpi, filtered and used to infect indicator HeLa P4.2 reporter cells. Equal amounts of virus determined by p24 quantification were used. β-galactosidase production was assessed by a colorimetric assay based on cleavage of CPRG. The data are means of three independent experiments carried out in triplicate. Normalization was performed as in **B.** P = 0.001. Error bars represent SEM. **, P<0.01.

We used the gentle shaking culture conditions to study differences between cell-free and cell-to-cell HIV-1 transmission. HIV-1-infected and control Jurkat T cells were cultured at 37°C under static or gentle shaking conditions and HIV-1 replication was compared. Viral replication was followed by determining the fraction of HIV-1-infected cells in the T cell cultures by measuring the percentage of Gag p24+ cells (Figure 1A). In static cultures viral replication resulted in increased appearance of Gag p24+ cells over the course of infection, reaching 42% infected Jurkat T cells by 7 days post-infection (dpi) while only 2% of the cells were Gag p24+ in the shaking cultures (Figure 1A). The HIV-1 yield in the supernatants was estimated by determining the HIV-1 p24 content by ELISA. At 7 dpi the virus yield was 10-fold higher in static than in shaken cultures (Figure 1B).

To determine the infectivity of viral particles, supernatants of the two culture conditions were recovered and virus infectivity was determined on HeLa p4.2 indicator cells. Equal amounts of virus determined by p24 quantification were used. Strikingly, the infectivity of cell-free viruses in the supernatants of shaken cultures was dramatically reduced, showing an approximately 10-fold reduction when compared with the infectivity of viruses from static cultures (Figure 1C). These results show that viruses produced by T cells cultured under gentle shaking conditions are not fully functional and suggest that the reduction of HIV-1 replication in these cells results from both prevention of cell-to-cell virus transmission as well as the loss of cell-free virus infectivity.

### Reduction of cell-free particle infectivity correlates with loss of HIV-1 envelope protein

**Figure 2:**
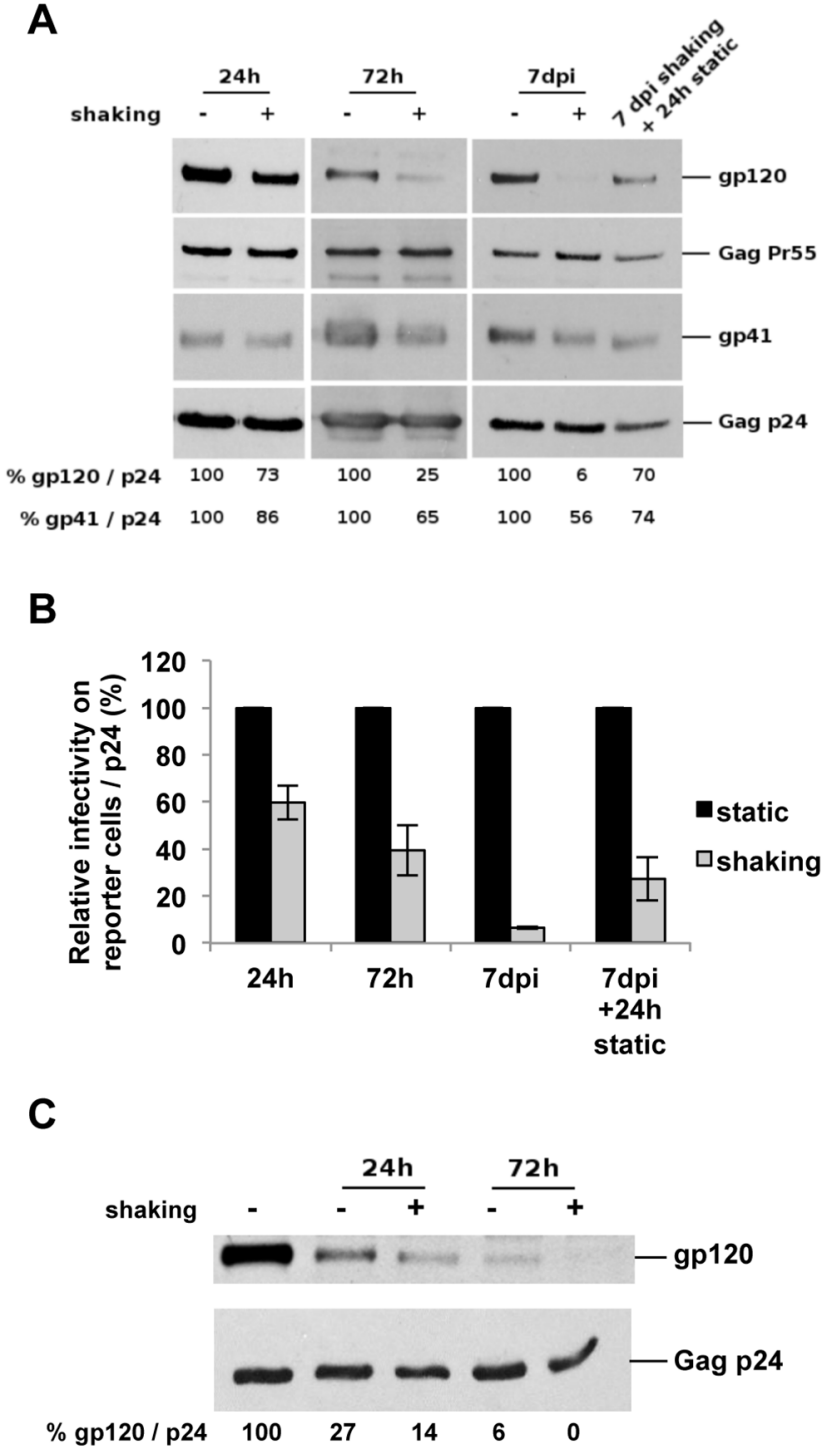
Loss of HIV-1 envelope protein correlates with loss of infectivity in cell-free particles produced by cells cultured under shaking conditions. **A.** Quantification of viral proteins from particles recovered in supernatants of static and shaking HIV-1-infected T-cell cultures. Purified viruses from supernatants of HIV-1-infected cells were recovered and concentrated by ultracentrifugation after 24 h, 72 h and 7 days of culture, as well as after 7 days under shaking conditions followed by an additional static culture of 24 h. These purified viruses were then analyzed using antibodies against the HIV-1 Gag and envelope proteins. The envelope levels, indicated under the western blot, were estimated from the intensity of the signals on western blots using the ImageJ software and calculated as gp120/p24 and as gp41/p24 ratios. Results of one representative experiment out of four independent performed are shown. **B. Infectivity of HIV-1 particles produced by static and shaking T-cell cultures.** Aliquots of viral supernatants analyzed in **A** were used to infect indicator HeLa P4.2 reporter cells with equal amounts of virus determined by p24 quantification. The infectivity of these viruses was determined as described in Figure 1C. The data are means of two independent experiments carried out in triplicate. The values were normalized taking as 100% the average value obtained for the static culture at each time point. Statistical analysis were performed on raw data. P = 0.015 for 24 h. P = 0.002 for 72 h. P = 0.002 for 7 dpi. P = 0.002 for 7 dpi+24 h static culture. Error bars represent SEM. **C. Quantification of viral proteins from particles incubated without cells under static and shaking culture conditions.** Equal amounts of purified viruses from supernatants of HIV-1-infected cells were incubated at 37°C during 24 h or 72 h. Analysis and quantification were performed as in A. The envelope levels were estimated from the intensity of the signals on western blots using the ImageJ software and calculated as gp120/p24 ratios.

In order to understand the loss of virus infectivity of particles produced by T cells cultured under shaking conditions, we examined the virion protein composition. HIV-1 particles produced by static (shaking −) and shaken (shaking +) T-cell cultures were analyzed by western blot (Figure 2). Ten million cells were infected at an MOI of 0.001, cultivated for 3 days and then divided for static or shaking culture. Supernatants containing viral particles produced during the course of infection were recovered. Purified viral particles were normalized by anti-p24 ELISA and equivalent amounts were analyzed. The levels of the HIV-1 proteins gp120, gp41, Gag Pr55 and Gag p24 were estimated using specific antibodies. HIV-1 particles recovered in supernatants of shaken T-cell cultures showed lower levels of the HIV-1 envelope gp120 and gp41 proteins than particles produced under static conditions (Figure 2A). The ratio of HIV-1 envelope protein (gp120 or gp41 subunit) over Gag protein levels, from viruses produced under static conditions was considered as 100%. Parallel cultures maintained under shaking conditions showed differences already after 24 hours (h) of culture. HIV-1 virions produced by these cultures showed 27% less gp120 compared with the particles from static cultures. Similarly, a 14% reduction of gp41 was observed. The loss of envelope proteins from viral particles produced by cells under shaking conditions after 72 h was higher (75% for gp120 and 35% for gp41). HIV-1 particles produced by cell cultures under these gentle shaking conditions for 7 days almost completely lost the gp120 protein and a reduction of about half of the gp41 protein subunit was also observed (94% reduction for gp120 and 44% reduction for gp41).

When cultures shaken for 7 days were cultivated for an additional 24 h under static conditions, the particles produced showed a recovery of the gp120 and gp41 contents compared to particles produced by the mobile T-cell cultures. This indicates that shaking did not cause a permanent defect on the producer cells. The reduction of gp120 and gp41 strongly correlated with the reduction of particle infectivity (Figure 2B). A reduction of 40%, 60% and 94% infectivity was observed after 24 h, 72 h and 7 days of culture under shaking conditions respectively. Strikingly, the recovery of the HIV-1 gp120 and gp41 protein levels observed after an additional 24 h of culture under static conditions correlated with a 4-fold increase in infectivity of these particles compared to the infectivity of those produced by cells cultured 7 days under shaking conditions (Figure 2B).

In order to determine if shaking conditions result in the reduction of gp120 of purified HIV-1 particles, cell-free particles were incubated at 37°C under the two culture conditions and gp120 levels were analyzed (Fig. 2C). Viruses incubated without cells under shaking conditions lost half of the amount of gp120 after 24 h and almost completely lost the gp120 glycoprotein at 72 h compared to those under static conditions (Fig. 2C). It is important to underline that levels of Gag p24 protein remained comparable in all conditions (Fig. 2C).

These results indicate that the alteration of the function and the composition of HIV-1 particles produced by the mobile lymphocytes very likely contribute to the poor efficiency of HIV-1 replication in shaken T-cell cultures.

### HIV-1 envelope expressed on the plasma membrane of T cells cultured under shaking conditions is not lost

**Figure 3:**
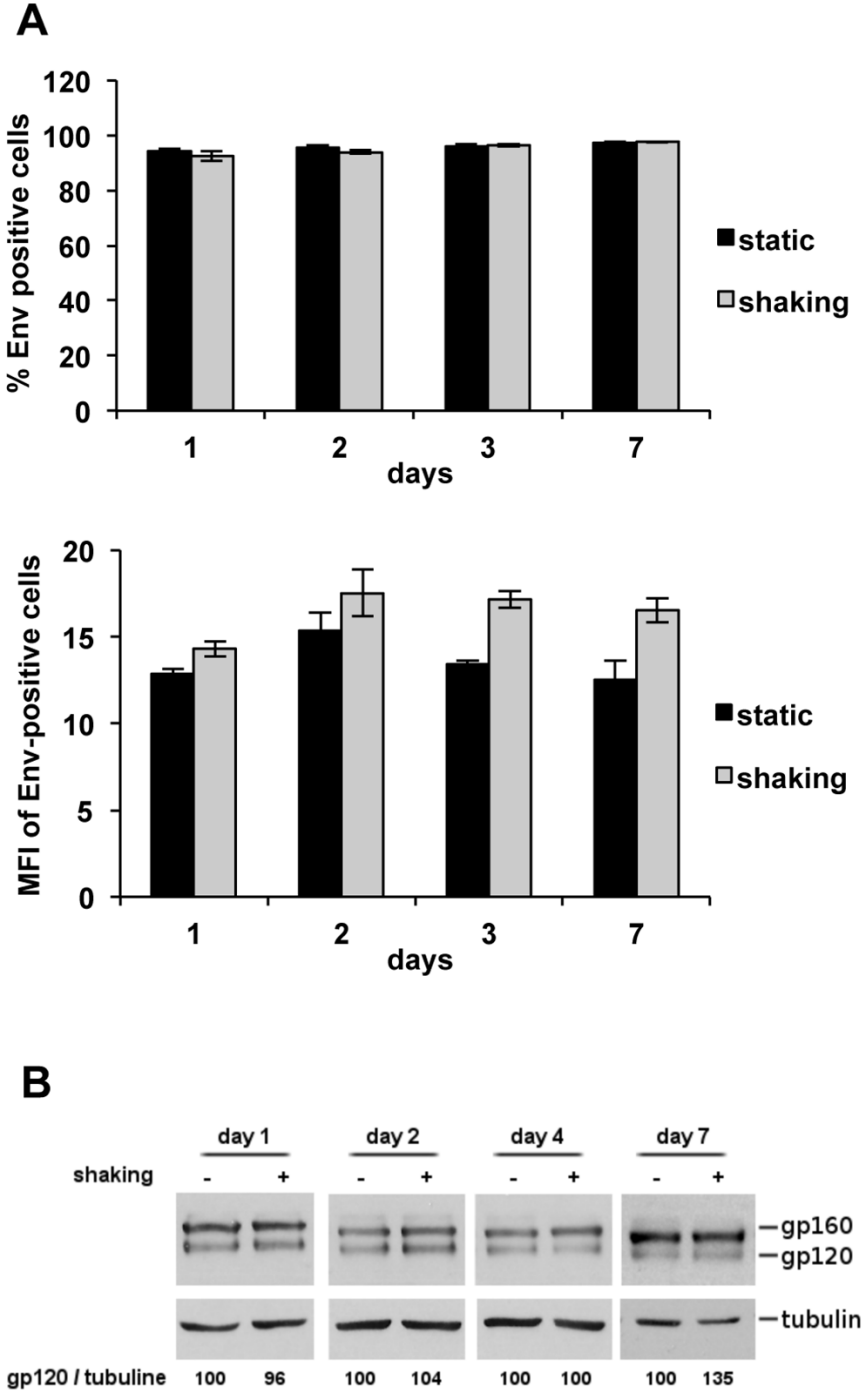
Stability of HIV-1 envelope expressed on the plasma membrane of T cells cultured under shaking conditions. **A.** Cell-surface level of HIV-1 envelope on CEM-213env1 cells cultured under static or shaking conditions. The T-cell line CEM-213env1 stably expressing the HIV-1 envelope glycoprotein was cultured for up to 7 days under static or shaking conditions. The cell-surface levels of the HIV-1 S envelope were determined by flow cytometry analysis using the anti-Env 5F7 antibodies. Percentage of Env-positive cells are shown in the top pannel and mean of fluorescence intensity (MFI) are shown in the bottom pannel. Two independent experiments were carried out in triplicate. Error bars represent standard deviations. **B. Western blot analysis and quantification of protein extracts of static and shaking CEM-213env1 T-cell cultures.** Equal amounts of total proteins from cell lysates recovered 1, 2, 4, and 7 days under static or shaking conditions were analyzed using antibodies against the HIV-1 envelope proteins. The envelope levels were estimated from the intensity of the signals on western blots using the ImageJ software and calculated as gp120/tubulin ratios. The results are from one representative experiment out of two independent experiments carried out in duplicate.

We attempted to determine the levels of envelope glycoprotein Env at the cell surface of HIV-1-infected T cells cultured under shaking conditions, but it was impossible to compare a similar number of Gag+ cells, since the infection levels of mobile lymphocytes were considerably lower than those of cells in static cultures. Therefore, to study the stability of HIV-1 Env after longer culture times, we analyzed the effect of shaking on the HIV-1 Env glycoprotein, using the T-cell line CEM-213env1 stably expressing the HIV-1 envelope glycoprotein [9]. CEM-213env1 cells were cultured under static or shaking conditions, the level of HIV-1 envelope was measured at the cell surface by flow cytometry analysis and the total amount of proteins was analyzed by western blot (Figure 3). Figure 3A shows no difference in the HIV-1 envelope level at the plasma membrane in CEM-213env1 cells cultured under static or shaking conditions after up to 7 days of culture. No difference was observed on both the percentage of Env-positive cells (Figure 3A top pannel) or the mean of fluorescence intensity (MFI; Figure 3A bottom pannel). These observations were confirmed by western blot analysis (Figure 3B). These results show that shaking culture does not affect the plasma membrane envelope levels of CEM cells expressing the HIV-1/213 envelope.

## Discussion and Conclusion

Our results indicate that the alteration of the function and the composition of HIV-1 particles produced under shaking conditions contributes to the poor efficiency of HIV-1 replication in these cultures. Since this system is widely accepted by the HIV-1 community to study differences between cell-free and cell-to-cell HIV-1 transmission, these results should be taken into account when working on HIV-1 transmission.

Gp120 shedding has been shown to vary between HIV-1 strains and a significant temperature-dependent shedding of gp120 from NL4-3 virions was observed [10]. Our studies were carried out only with the NL4.3 strain that is the main strain used to study HIV-1 transmission under shaking culture conditions. Future work must be performed to assess whether a similar effect is seen with other strains, including primary isolates and R5 strains. If shaking does not lead to shedding of the gp120 subunits, the culture method may still be meaningful with other isolates. Presently, we do not have an explanation for the reduction we observed on the levels of gp41 after culture under shaking conditions.

A viral particle holds only 7 to 14 Env spikes on its surface [11], whereas an infected cell presents large clusters of Env spikes at assembly sites all over the membrane [12]. This may explain why envelope proteins are lost from the surface of viral particles and not from the plasma membrane of Env-expressing cells. Besides, the envelope protein is renewed in the cells by continuous synthesis whereas the amount incorporated in viral particles is permanent. It was reported that shaking does not affect cell viability, metabolism and surface molecule levels such as CD4, CXCR4, MHC-I, CD11a, CD18, ICAM-1, ICAM-3 [4]. Our results show that shaking culture does not affect the plasma membrane envelope levels of CEM cells expressing the HIV-1/213 envelope on their surface. That shedding does not occur with other cell-expressed HIV-1 envelope strains would need to be analyzed in future studies.

Finally, even though we focused on cell-to-cell transfer and spike integrity in this study, it is possible that shaking might affect other aspects of cell cultures that impede viral spread, such as the ability to establish multivalent virus-cell contacts and post-entry passage through the cellular cytoskeleton.
